# IRIS-EDA: An integrated RNA-Seq interpretation system for gene expression data analysis

**DOI:** 10.1371/journal.pcbi.1006792

**Published:** 2019-02-14

**Authors:** Brandon Monier, Adam McDermaid, Cankun Wang, Jing Zhao, Allison Miller, Anne Fennell, Qin Ma

**Affiliations:** 1 Institute for Genomic Diversity, Cornell University, Ithaca, NY, United States of America; 2 Department of Biology & Microbiology, South Dakota State University, Brookings, SD, United States of America; 3 Department of Agronomy, Horticulture, and Plant Science, BioSNTR, South Dakota State University, Brookings, SD, United States of America; 4 Department of Biomedical Informatics, College of Medicine, The Ohio State University, Columbus, OH, United States of America; 5 Department of Biology, Saint Louis University, St. Louis, MO, United States of America; 6 Donald Danforth Plant Science Center, St. Louis, MO, United States of America; Johns Hopkins University, UNITED STATES

## Abstract

Next-Generation Sequencing has made available substantial amounts of large-scale Omics data, providing unprecedented opportunities to understand complex biological systems. Specifically, the value of RNA-Sequencing (RNA-Seq) data has been confirmed in inferring how gene regulatory systems will respond under various conditions (bulk data) or cell types (single-cell data). RNA-Seq can generate genome-scale gene expression profiles that can be further analyzed using correlation analysis, co-expression analysis, clustering, differential gene expression (DGE), among many other studies. While these analyses can provide invaluable information related to gene expression, integration and interpretation of the results can prove challenging. Here we present a tool called IRIS-EDA, which is a Shiny web server for expression data analysis. It provides a straightforward and user-friendly platform for performing numerous computational analyses on user-provided RNA-Seq or Single-cell RNA-Seq (scRNA-Seq) data. Specifically, three commonly used R packages (edgeR, DESeq2, and limma) are implemented in the DGE analysis with seven unique experimental design functionalities, including a user-specified design matrix option. Seven discovery-driven methods and tools (correlation analysis, heatmap, clustering, biclustering, Principal Component Analysis (PCA), Multidimensional Scaling (MDS), and t-distributed Stochastic Neighbor Embedding (t-SNE)) are provided for gene expression exploration which is useful for designing experimental hypotheses and determining key factors for comprehensive DGE analysis. Furthermore, this platform integrates seven visualization tools in a highly interactive manner, for improved interpretation of the analyses. It is noteworthy that, for the first time, IRIS-EDA provides a framework to expedite submission of data and results to NCBI’s Gene Expression Omnibus following the FAIR (Findable, Accessible, Interoperable and Reusable) Data Principles. IRIS-EDA is freely available at http://bmbl.sdstate.edu/IRIS/.

This is a *PLOS Computational Biology* Software paper.

## Introduction

Advanced computational tools with appropriate experimental designs and interactive interface are needed to build integrated models of biological systems and devise deliverable strategies to prevent or treat disease [1–3]. RNA-Seq has created vast amounts of gene expression data and the demand for data analysis and interpretation is significant [4]. Analysis of the gene expression data is facilitated by computational experience in appropriately designing the methods and experiments and conducting the analysis processes using one of many computing languages. This creates an obstacle for users with limited computational experience who want to analyze their RNA-Seq studies; thus there is an increased need for easy-to-use interactive expression analyses and results visualization [5].

While a wide variety of computational methods can be applied to expression data to determine particular qualities of the data on a sample or cell level [6–13], differential gene expression (DGE) analysis is the most commonly used. It allows researchers to identify differentially expressed genes (DEGs) across two or more conditions and can provide a meaningful way to correlate differences in gene expression levels with phenotypic variation. Many tools have been developed and optimized, such as: DESeq [14], DESeq2 [15], edgeR [16], limma [17], Cuffdiff [18], Cuffdiff2 [19], sleuth [20], and many others. While there have been substantial efforts in DGE analysis and visualization of DGE results [21–28], numerous pitfalls and bottlenecks persist, including challenges with experimental design, a need for comprehensive integrated discovery-driven analyses and DGE tools, and the lack of functionalities and interactivity related to visualizing the analysis results.

**Fig 1:**
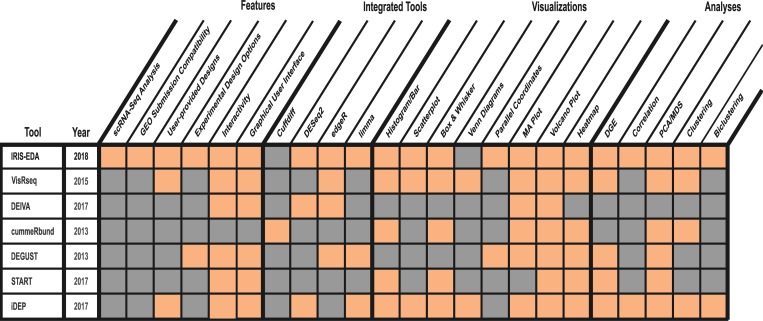
IRIS-EDA integrated functions. Comparison of IRIS-EDA and six other DGE analyses and visualization tools regarding available features, integrated tools, visualizations, and analyses.

To address these bottlenecks, we have created IRIS-EDA, which is an **I**nteractive **R**NA-Seq **I**nterpretation **S**ystem for **E**xpression **D**ata **A**nalysis. It provides a user-friendly interactive platform to analyze gene expression data comprehensively and to generate interactive summary visualizations readily. In contrast to other analysis platforms, IRIS-EDA provides the user with a more comprehensive and multi-level analysis platform. IRIS-EDA outperforms other tools in several critical areas related to efficiency and versatility offering: 1) Single-cell and bulk RNA-Seq analysis capabilities, 2) GEO submission compatibility, 3) seven useful discovery-driven and DGE analyses, 4) seven experimental design approaches through three integrated tools for DGE analysis, and 5) seven interactive visualizations (Fig 1).

Specifically, IRIS-EDA provides comprehensive RNA-Seq data processing and analysis in a seamless workflow. This investigative approach uses expression quality control and discovery-driven analyses integrated with DGE analysis through one of the three widely used R packages, *DESeq2*, *edgeR*, and *limma*, all of which have demonstrated capacities for expression data analysis [29]. It provides users with a choice of intuitive experimental design options (e.g., pairwise and factorial comparisons, main and grouped main effects, etc.), as well as, the option to upload a custom design matrix in the DGE analysis. IRIS-EDA includes numerous interactive visualizations for each analysis type, enabling users to gain an immediate global view of their data and results or download as a high-resolution static image for publications. For the first time, this tool implements a framework based on the FAIR Data Principles [30] to assist users with the submission of their data and results to NCBI’s Gene Expression Omnibus (GEO).

## Design and implementation

### Bulk and single-cell RNA-Seq analysis

IRIS-EDA was designed to provide a comprehensive platform for gene expression data analysis, which includes applicable analysis of both bulk and single-cell sequencing data. Single-cell RNA-Seq (scRNA-Seq) data analysis is a growing area of study within RNA-Seq analyses and can provide unique insights into gene expression patterns considering cell variations [31, 32]. The methods used for traditional DGE analysis have demonstrated applicability to scRNA-Seq DGE analysis when combined with proper filtering and DGE methods [32]. Thus, IRIS-EDA can facilitate discovery-driven and DGE analysis for scRNA-Seq data with few modifications. Namely, analysis of single-cell data can be appropriately carried out by using a stringent filter cutoff based on a default setting of transcripts per million (TPM) > 1, especially when combined with either edgeR or limma, which have both been shown to have high performance on scRNA-Seq data [32]. For particular types of scRNA-Seq data that expect overall low expression levels, such as 10X single-cell data, a different approach is provided to account for the differences. In particular, DESeq2 normalization methods are used in conjunction with no filtering of genes to provide the most reliable analysis results [33]. More details regarding the analysis of scRNA-Seq data can be found in the *Single-cell RNA-Seq* section of *S1 Text*.

### Required inputs

IRIS-EDA requires two or three user-provided input files, depending on the type of data used: (1) a gene expression estimation matrix (EEM, also referred to as read count data), (2) a condition matrix with factor levels corresponding to the provided samples in the EEM, and (3) a gene length matrix indicating the base-pair length of each gene to be used for filtering of scRNA-Seq data only. When uploading data, users will select gene expression data type: either bulk or single-cell RNA-Seq data. If using scRNA-Seq data, the additional requirement for gene length matrix will be shown on the web server. Also, default parameterizations for optimized analysis for single-cell data will be populated throughout the server. Methods to obtain gene lengths from GFF/GTF/GFF3 annotation files can be found in the *Single-cell RNA-Seq* section of *S1 Text*.

After submitting the required inputs, one of the three normalization approaches can be selected, or users can choose not to normalize the data. The three normalization methods available in IRIS-EDA are the normal log transformation, regularized log transformation, and variance stabilizing transformation. The normal log transformation uses a base-2 log function to normalize the expression for each gene. Doing so improved the expression distribution visualizations, particularly for sparse expression matrices where the large number of zeros can lead to little information collected from non-transformed plots. The regularized log transformation provides a method to minimize the differences between samples with small gene counts and regularizes based on library size [15]. The regularized log transformation method is most useful for datasets where library sizes do not vary greatly. The variance stabilizing transformation also normalizes by library size and provides an expression matrix that is roughly homoscedastic [15]. For datasets with library sizes that vary greatly, the variance stabilizing transformation method would be most appropriate.

### Discovery-driven analyses

Discovery-driven analyses include tools and algorithms designed to provide an investigative approach of expression data, especially for the situation where users do not have a strong direction or hypothesis for their data analysis procedures. These algorithms assist users in analyzing and visualizing their EEM input information and discovering trends in their data that may provide additional hypotheses for downstream analyses. In particular, discovery-driven analyses can help users define a specific hypothesis within their RNA-Seq study, which can assist in development of experimental design methods for DGE analysis. Discovery-driven analyses processes available in IRIS-EDA include: sample correlation analysis and pairwise expression scatterplots, expression heatmaps, clustering, biclustering, principal component analysis, multidimensional scaling, t-distributed Stochastic Neighbor Embedding, and sample distance matrix. The figures generated through the discovery-driven analysis feature of IRIS-EDA are provided in an interactive manner, allowing users to select specific samples or pairwise comparisons to further evaluate. One such example is with the sample correlation analysis and pairwise scatterplots. Users can choose one cell of the sample correlation matrix corresponding to a comparison between two samples. This will display the pairwise scatterplot for that specific comparison. The user can then scroll over the scatterplot and display the gene ID for an indicated data point. A detailed example with more tutorial information will be shown in the Results section.

### Differential gene expression analysis

DGE analysis in IRIS-EDA is performed using one of three tools provided: *DESeq2* [14], *edgeR* [16], and *limma* [17]. These three tools were selected based on their widespread use in published RNA-Seq studies and reviews [28]. The default tool is *DESeq2*, based on independent evidence supporting its performance [29] and our RNA-Seq analysis experience, but users can also select one of the other two tools based on their own preference. There are other high-performing commonly-used DGE tools available; however, their compatibility with IRIS-EDA excludes their use in IRIS-EDA. For example, tools that do not utilize read count data, e.g., *Sleuth*, [20] or are not R-based, e.g., *Cuffdiff* [18], are not included due to compatibility issues.

In addition to the DGE tools, experimental design can also be specified by the user. The designs provided in IRIS-EDA include two-group (pairwise) comparisons, multiple factorial comparisons, classic interaction design, additive models for pairing or blocking of data, main effect testing (testing time-series data) and blocked main effect testing. IRIS-EDA provides additional flexibility for the instances when the user needs a design not already included in IRIS-EDA. Each of these methods has unique parameters to be specified by the user, typically including which factors are intended for analysis and which specific comparisons are required. After analyzing the data, IRIS-EDA provides an overview displaying the number of up- and down-regulated IDs for each indicated comparison, along with a histogram displaying this information. The results table is also available through IRIS-EDA, along with interactive MA and Volcano plots. Both of these plots allow users to compare DGE results metrics, such as log fold-change, mean expression, and adjusted p-value.

Similar to the figures generated in the Discovery-Driven Analysis section of IRIS-EDA, the plots in the DGE section are also highly interactive. Discovery-Driven Analysis features allow users to gain more specific information from their plots, including highlighting individual or regions of data points on the plot. These features highlight the corresponding row of the DGE results table, showing users gene information identifying them as outliers or falling within a certain region. Conversely, users can select specific gene IDs from the results table, resulting in the highlighting of that gene ID’s or set of gene IDs’ data points on the corresponding plot. This feature can be used to easily determine the relative location of specific genes or gene sets in the plot.

Results obtained from the DGE analysis section of IRIS-EDA are often not the end of the analysis procedures. Based on the information collected, users may choose to further investigate their expression data using additional analyses provided in the Discovery-Driven Analyses section, such as the clustering or biclustering. This feedback loop between DGE and Discovery-Driven analyses allows for supporting and complementing analyses to function in tandem, providing more comprehensive data interpretation.

### IRIS-EDA outputs

IRIS-EDA provides users with methods for extracting content based on discovery-driven and DGE analyses. All figures in the Quality Control, Discovery-Driven Analysis, and DGE Analysis sections have the option for users to download as a static image in PDF or PNG format. Additionally, all tables in the DGE Analysis section are downloadable as CSV files, with the final results table being downloaded in its entirety or filtered based on user-provided or default-adjusted p-value and log fold-change cutoffs. The DGE Analysis results can also be used for functional enrichment analysis, with detailed instructions included in this tab and in S1 Text S7.3. As part of the clustering and biclustering analyses, users can also download a list of gene IDs contained within the specified cluster.

### GEO submission and FAIR Data principles compatibility

Many users are also interested in submitting their RNA-Seq data to a public repository for accessibility, but this process can be tedious and troublesome. NCBI’s GEO database has specific requirements related to the data, results, and accompanying metadata file. To assist users in their preparation of documents for GEO submission, IRIS-EDA offers an optional GEO page. In following with the standard set forth by the FAIR Data Principles [30], this page asks users to provide a limited amount of information that will be used, along with the previously provided condition matrix information, to populate the metadata file required for GEO submission. This populated metadata file will then be available for download with reformatted processed data files extracted from the EEM. These two pieces of information can later be submitted with the original raw FASTQ-formatted RNA-Seq data to the GEO submission page. More detailed information regarding the usage of the GEO capabilities of IRIS-EDA can be found in the *GEO Usage* section of *S1 Text*.

## Results: An application example using scRNA-Seq data

To demonstrate the effectiveness of IRIS-EDA, we analyzed a scRNA-Seq dataset consisting of human tissue cells from various cell types. The expression data was taken from Yan, et al. [34]. The raw counts file was uploaded as the example scRNA-Seq data available on the IRIS-EDA server. All requisite information, including sample information and gene lengths are automatically provided using the example datasets. The scRNA-Seq example dataset is composed of 90 cells. While this may not be entirely representative for the ever-increasing size of scRNA-Seq datasets, this will be used for example purposes due to size limitations. Users interested in analyzing larger datasets (1000+ cells) should refer to the S1 Text S9 for information related to the optimized use of the IRIS-EDA server for this purpose, as well as how to access an example dataset of this size.

### Gene expression data quality control

After data upload, the three input files are first analyzed by IRIS-EDA quality control. Input data quality is evaluated using boxplots and histograms of the read count distributions. The purpose of the quality control process is to enable exploration of the submitted data and to verify that there are no unexpected or unexplainable abnormalities in the data, such as low total read counts or individual samples displaying strange distribution behavior. Based on the scRNA-Seq filtration method of TPM > 1, 78 out of the original 3,679 genes were filtered, leaving a total of 3,601 genes. This process is conducted to reduce the false-positive rate experienced in analyses related to scRNA-Seq data. Following data upload and initial quality check, users can continue on to the Discovery-Driven Analyses section of IRIS-EDA, which is broken down further into five subsections.

#### Correlation analyses

**Fig 2:**
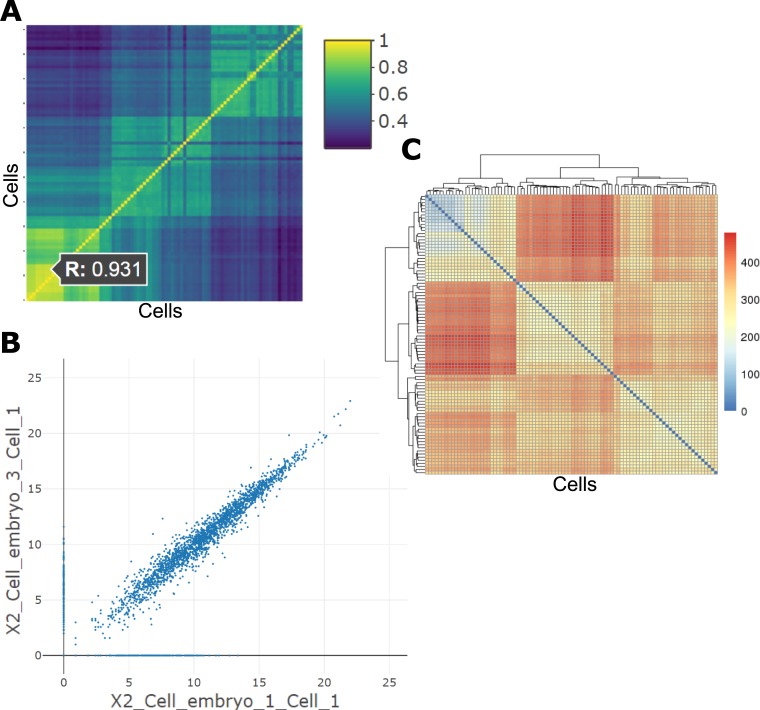
Correlation analyses. (A) Interactive correlation heatmap generated from single-cell gene expression data; (B) Scatterplot generated by selected a cell in the interactive correlation heatmap; (C) Sample distance matrix showing Euclidean distances between samples, along with hierarchical clustering.

The analyses under the “Correlation” tab of the Discovery-Driven Analyses provide a pairwise sample Pearson correlation value through an interactive heatmap. Selecting a cell in this heatmap generates the indicated pairwise scatterplot of gene expression values. In this example, two two-cell embryo samples are chosen, indicating a correlation value of 0.931, which is relatively high compared to the correlation observed in the dark blue cells, such as the morulae cells compared with the late blastocyst cells (Fig 2A). The scatterplot that is generated from selecting this sample comparison shows high clustering of data points along the diagonal, indicating a high similarity between these two samples across all gene expression levels (Fig 2B). The sample distance matrix also shows supporting information is this comparison, in that the multi-cell embryo samples cluster separately from the late blastocyst samples (Fig 2C).

#### Principal component analysis, multidimensional scaling, and t-distributed Stochastic Neighbor Embedding

**Fig 3:**
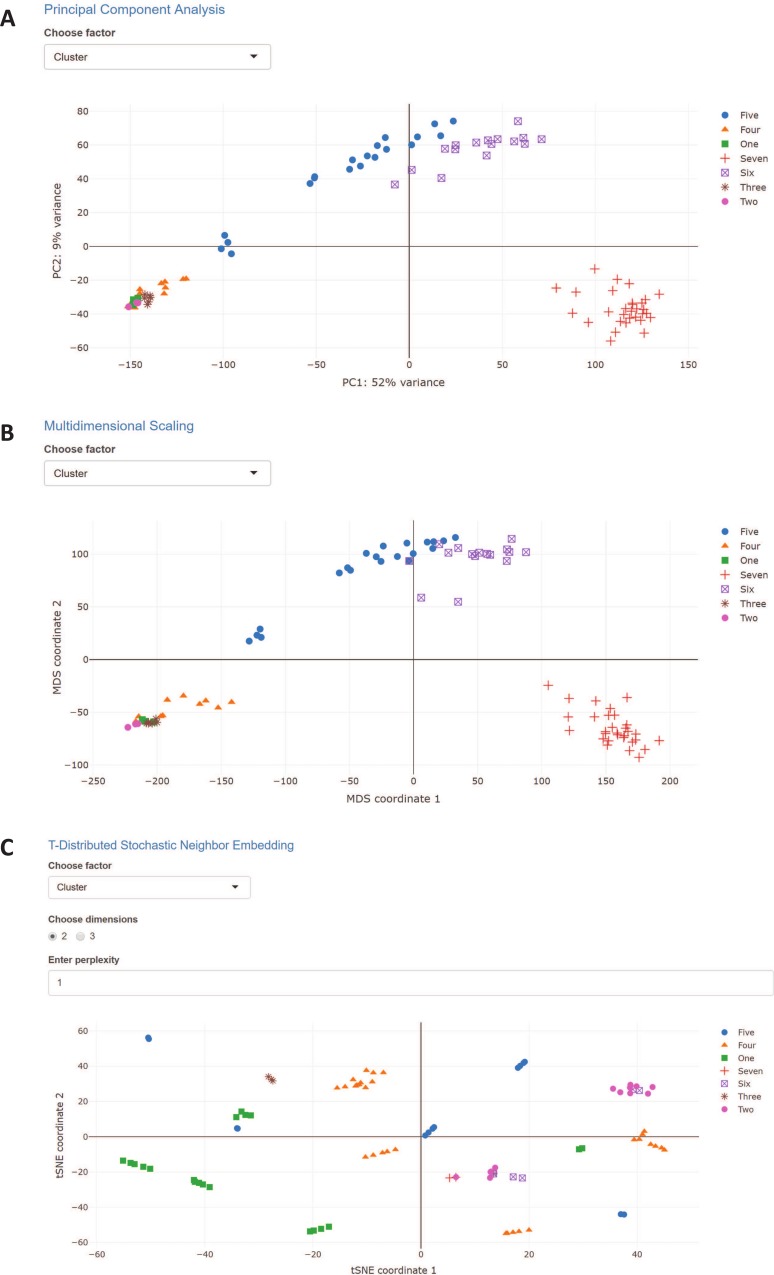
Principal component analysis, multidimensional scaling, and t-distributed Stochastic Neighbor Embedding. (A) PCA plot showing the first two principal components; (B) MDS plot showing the first two MDS coordinates; and (C) t-SNE plot showing the first two t-SNE coordinates.

PCA, MDS, and t-SNE provide linear, non-linear, and non-parametric transformations, respectively, of the gene expression vectors represented by each sample for dimension reduction. The transformations are then commonly plotted as scatterplots by the first two principal components representing the most variance between samples. Where the most variance is observed in the first and second components, particular clusters of cells appear to group together, indicating high similarity about that transformation. In the scRNA-Seq example, clusters Five and Six and clusters One, Two, Three, and Four group together closely in both the PCA (Fig 3A) and MDS (Fig 3B) plots, while cluster Seven is quite isolated. This shows a high level of difference between the late blastocyst samples and other samples. The t-SNE feature allows for visualization of either two or three dimensions, with the three-dimensional plot allowing for rotation of the axes. The three-dimensional plot of the scRNA-Seq example data shows mostly clustering of similar clusters, except a single instance of clusters Seven, Six, and Two grouping together.

#### Clustering

**Fig 4:**
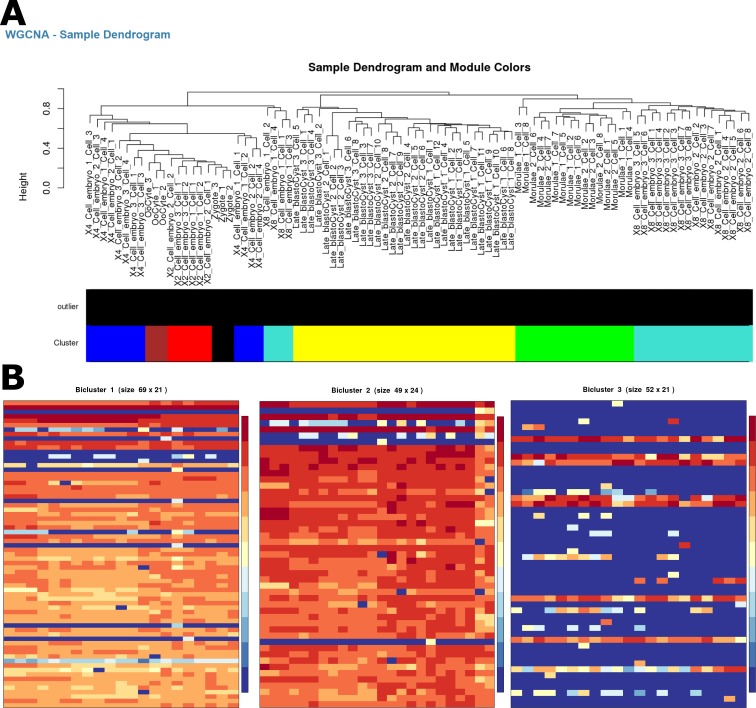
Clustering and biclustering. (A) Sample dendrogram and color bar representing optimized identified clusters for the WGCNA method of clustering on the scRNA-Seq example data. The dendrogram shows the 2- and 4-cell samples clustering together, with the Late Blastocysts forming a unique cluster. (B) The first three biclusters were generated using QUBIC on the IRIS-EDA server. The first two biclusters (69 and 49 genes) show the grouping of Oocyte, Zygote, and 2- and 4-cell samples and Oocytes, Zygote, and 2-, 4-, and 8-cell samples, respectively. The third bicluster (52 genes) separates the Late Blastocysts from the other samples. These three biclusters demonstrate the expression similarity between the Oocyte, Zygote, and multi-cell samples relative to the Late Blastocyst samples over numerous gene sets.

Clustering of samples based on gene expression can provide helpful insight to group samples and conditions with similar expression level across all genes. For scRNA-Seq data, clustering can provide information related to cell types. While clustering methods alone cannot lead to full cell-type prediction, they can help support other cell-type prediction methods. State-of-the-art cell-type prediction methods involve at least two steps, one of which is a clustering approach [35]. In IRIS-EDA, three clustering methods are provided: Weighted Gene Co-expression Network Analysis (WGCNA) [36], k-medoids [37], and the Markov Clustering Algorithm (MCL) [38]. WGCNA, k-medoids, and MCL represent the highest performing clustering methods from hierarchical, representative, and graph-based clustering approaches, with WGCNA being the highest overall performer [9]. Because of this, we selected WGCNA for use in the example, generating results related to the hierarchical clustering of samples (Fig 4A). In this WGCNA analysis, the 2-cell and 4-cell samples cluster together quite closely, and the 8-cell samples were distributed throughout the remaining cell types.

#### Biclustering

Biclustering can group together subsets of the expression profile, indicating genes that have high expression similarity in only a subset of cells. Heatmaps for the first three biclusters are shown in Fig 4B, with the first two showing expression similarities for the Oocyte, Zygote, and Embryo cells, while the third cluster shows high homogeneity for the late blastocyst cells. This information is also supported in the PCA and MDS analyses.

### Differential gene expression analysis

#### Experimental design

For the purpose of analyzing the example scRNA-Seq data, we will be using the basic two group comparison design, which looks for differences between selected clusters. Based on the information in the Discovery-Driven Analyses section, we know the samples in cluster Seven appear different than the other samples. Because of this, the factor levels chosen for comparison are all comparisons involving cluster Seven.

#### DGE overview

**Fig 5:**
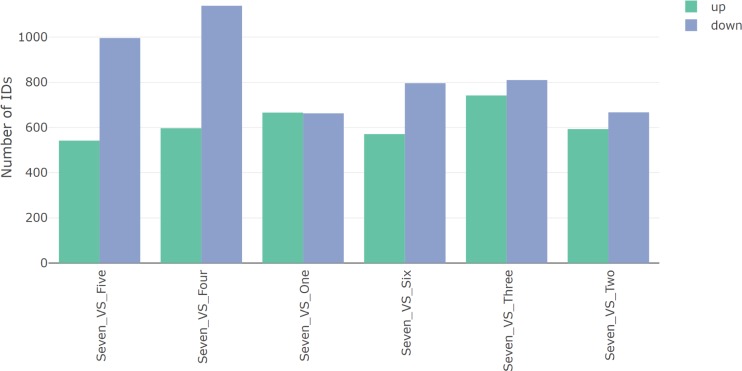
DGE overview. An overview of the number of DEGs determined using DESeq2 on the IRIS-EDA server. Cell-type Seven is compared against the other six cell types based on the number of up- and down-regulated genes. The Seven and Four comparison shows the highest number of DEGs of all comparisons, followed by Seven and Five comparison. The other four comparisons show similar numbers of DEGs, with all comparisons showing at least as many down-regulated genes as up.

The Overview tab of the DGE Analysis section in IRIS-EDA provides basic information related to the number of DEGs in the selected comparisons, specifically the number of up- and down-regulated genes. This information is provided as a table and as a bar plot (Fig 5). The single-cell example data, with pairwise comparisons relative to cluster Seven, show most comparisons having more down-regulated genes. This indicates cluster Seven has higher expression levels of a large number of genes compared to the other clusters. In particular, the Seven and Four comparison has the highest number of down-regulated DEGs and the highest number of DEGs overall.

#### DGE plots

**Fig 6:**
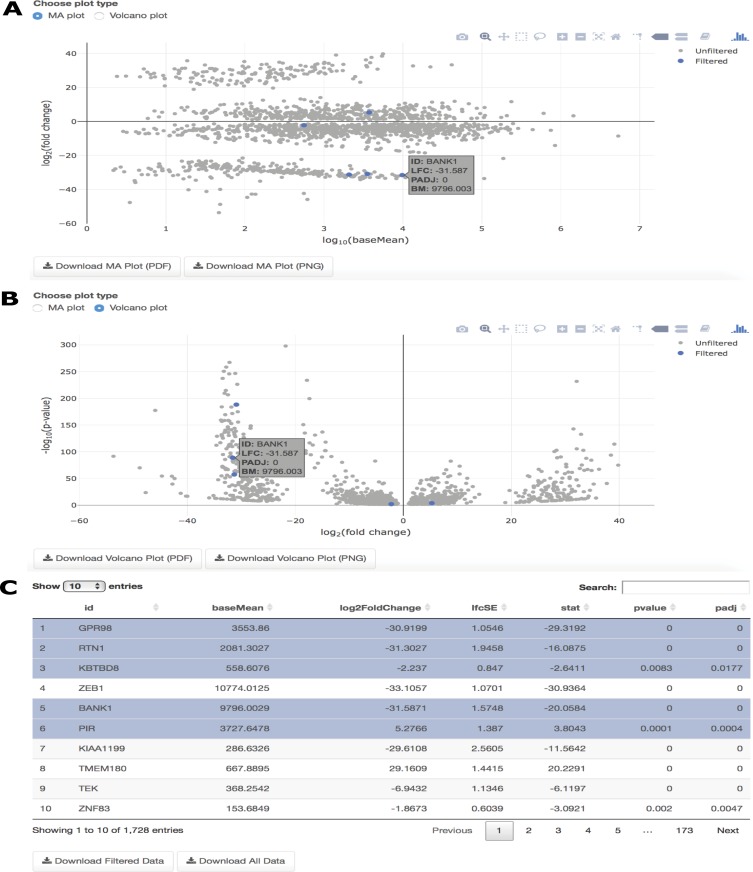
DGE overview. (A) MA plot for the Seven and Four cluster comparison with particular genes highlighted in the results table and corresponding location in the figure; (B) Volcano plot for the Seven and Four cluster comparison with particular genes highlighted in the results table and corresponding location in the figure; (C) The searchable, interactive table corresponding to both the MA plot and Volcano plot, showing results of the DGE analysis from the user-selected DGE tool.

Two interactive plots are provided following DGE analysis on IRIS-EDA. Both are accompanied by a linked table, which highlights the results information for a selected gene in the figure or highlights the gene in the figure corresponding to the selected gene from the table. Since the Seven vs. Four cluster comparison shows the highest number of DEGs from the DGE Overview table, this comparison seems like an interesting choice to explore further using the DGE plots. Both the MA plot and Volcano plot show features of potential genes of interest. The MA plot gives a visual representation of mean expression compared with log fold-change for a selected comparison, while the Volcano plot compares log fold-change with adjusted p-values. BANK1, which is associated with calcium binding in the central nervous system, is highly differentially expressed between the late blastocyst and 4-cell embryo samples. This high absolute log fold-change is shown in both the MA and Volcano plots (Fig 6A & 6B) by selecting BANK1 in the interactive table (Fig 6C).

## Availability and future directions

The Shiny open-source tool can be accessed through the direct URL bmbl.sdstate.edu/IRIS/ or can be loaded locally using basic R code loaded through GitHub (https://github.com/btmonier/iris). The tutorial found on the server and in *S1 Text* provides a comprehensive explanation of all features within the IRIS-EDA tool, including descriptions of how to optimally use each feature. Descriptions of the interpretations for each analysis can also be found in this document.

In future iterations of this tool, we plan to expand the scope of analyses that IRIS-EDA can cover. Analyses such as functional enrichment, motif prediction [39], and various other network analyses have the potential to provide further insight into expression data. Thus, the inclusion of these analyses would benefit a certain segment of researchers. Additionally, we plan to explore the implementation of IRIS-EDA in the Galaxy platform [40]. This implementation would allow for an even broader base of users for this tool.

As demonstrated through the discussion of methods and demonstration using scRNA-Seq data, IRIS-EDA provides a method for comprehensive analysis of expression data. It is our hope that this tool will have a substantial impact on researchers aiming to explore and analyze both bulk and single-cell RNA-Seq data.

## Supporting information

S1 Text IRIS-EDA supplementary material. Detailed tutorial for the IRIS-EDA web server.
(PDF)
